# Putative Photosensitivity-Associated Sexual Dimorphism in Compound Eye Structure of *Lymantria xylina* (Lepidoptera: Erebidae)

**DOI:** 10.3390/insects16111122

**Published:** 2025-11-01

**Authors:** Hui Jiang, Tao Ni, Siyi Liu, Meng Wang, Jialing Zheng, Baode Wang, Songqing Wu, Feiping Zhang, Rong Wang

**Affiliations:** 1 State Key Laboratory of Agricultural and Forestry Biosecurity, College of Forestry, Fujian Agriculture and Forestry University, Fuzhou 350002, China; 17720685880@163.com (H.J.); 1220429010@fafu.edu.cn (T.N.); liusiyipippa@163.com (S.L.);; 2 US Department of Agriculture, Animal and Plant Health Inspection Service, S&T, Forest Pest Methods Laboratory, Buzzards Bay, MA 02542, USA; baode.wang@usda.gov

**Keywords:** invasive pests, compound eyes, visual system, ultrastructure

## Abstract

**Simple Summary:**

*Lymantria xylina* is not only a serious defoliator pest but also a close relative of the spongy moth, *L. dispar*, drawing widespread international attention. Using scanning electron microscopy technology, sexual dimorphism in compound eye morphology was examined, with males exhibiting significantly larger compound eyes, larger facet areas, larger facet perimeters, and a greater total number of facets than females. Although transmission electron microscopy reveals that the fundamental ommatidium structure, comprising a cornea, a crystalline cone, and a rhabdom with eight retinular cells, remains consistent, males possess thinner corneas and elongated crystalline cones. Multiple differences in compound eye structures indicate that males may exhibit enhanced sensitivity and adaptability to light compared to females. However, females achieve adaptability through the regulation of pigment granule translocation in response to changing light conditions.

**Abstract:**

*Lymantria xylina* is a major pest in coastal casuarina shelterbelts and a species subject to quarantine regulations by countries to which it is non-native. Phototaxis is fundamental to the insect’s surveillance and risk assessment analysis, and it exhibits pronounced sexual dimorphism in compound eye structure. This dimorphism was investigated using scanning and transmission electron microscopy. Males displayed significantly larger compound eyes, characterized by greater ommatidial areas and a higher total number of facets per eye compared to females. From the distal to proximal end, the ommatidium consists of the cornea, primary and secondary pigment cells, crystalline cones, retinula cells, a rhabdom bundle, and basal retinal cells (in a “7 + 1” arrangement). The internal ultrastructure of the ommatidia is similar in both sexes. However, males possess significantly thinner cornea and extremely elongated crystalline cones. Based on external morphology, both sexes generally exhibit a parallel-symmetrical compound eye form, minimizing optical asymmetry to optimize nocturnal vision. These differences are attributed to the distinct visual demands of males for mate-searching in low-light environments, while females, being more stationary, have reduced visual needs. Paraffin sections of *Lymantria xylina* compound eyes further revealed that, during dark adaptation, pigment granules aggregated within the crystalline cone region to enhance low-light capture. Conversely, following intense light stimulation, these granules translocated to the perinuclear region of photoreceptor cells, forming a light-shielding configuration.

## 1. Introduction

The evergreen shrub and tree species *Casuarina equisetifolia* is an important species for establishing shelter forests in subtropical coastal areas of China. It is well known for its resistance to drought, salt, and alkalies, and its ability to act as a windbreak and to stabilize sand [1,2]. In the southeastern coastal regions, over 160,000 hectares of casuarina plantations have been established [3]. To date, 155 pest species have been recorded on *C. equisetifolia*, with casuarina moth, *Lymantria xylina*, being the most significant defoliating pest in the southern coast of China [4,5].

The casuarina moth exhibits a univoltine life cycle. Its eggs remain in a diapause state for up to 9 months, hatching in spring (typically March–April). Larval development progresses through 6–7 instars before pupation occurs. Adults emerge from late May to June. Consequently, intensive defoliation is concentrated during the spring to early summer period [6]. The larvae can completely defoliate the branches and leaves of casuarina shelter forests during their outbreaks, threatening the ecological function and sustainable development of coastal forests [7]. As a typical lepidopteran pest in Lymantriidae, the casuarina moth feeds on around 424 trees and shrubs from 103 families [5,8]. The male of the species has strong flying abilities and is capable of searching for the sex pheromones released by the female over long distances to mate, while the female is less active [9]. It shares many similar biological characteristics with the spongy moth, *L. dispar*, including nocturnal activity patterns, males possessing strong flight capacity for long-distance female location, comparatively sedentary females, and characteristic phototactic behavior, highlighting the importance of controlling the spread worldwide [5,10,11]. The adults exhibit clear phototactic behavior, primarily engaging in feeding, mating, and other behaviors at night, making them a typical nocturnal insect. Males exhibit significantly stronger phototactic capabilities than females. Female moths primarily engage in short-range phototactic flights during early evening hours, whereas males demonstrate heightened activity during late-night hours. The field-captured male-to-female ratio in traps is approximately 322:1. While both sexes demonstrate peak attraction to light sources at 365 nm, laboratory-based behavioral assays revealed that merely 0.6% of females flew directly towards the light source, compared with up to 33.3% of males [9,12,13].

Phototaxis, a key characteristic of many nocturnal Lepidoptera insects [14], is notably influenced not only by environmental factors, including the wavelength and brightness of light sources, but also by the photosensitive structures of the insect’s compound eyes and the distribution of photoreceptor cells [14,15]. Insect behavior is shaped by the integration of neural action potentials and visual patterns. The compound eye, which serves as the primary organ for detecting light changes and object characteristics, plays a crucial role in the life history strategies of insects [16,17]. Insect compound eyes are principally categorized into apposition or superposition types. Apposition eyes, characteristic of diurnal insects, produce high-resolution images under bright illumination but exhibit low sensitivity under dim light conditions, In contrast, superposition eyes, commonly found in nocturnal moths such as *L. xylina*, enhance nocturnal light capture at the expense of reduced resolution [18].

The most basic optical unit of a compound eye is an ommatidium, and generally, the larger and more numerous the ommatidia, the stronger the insect’s visual abilities and the clearer the resulting image [19]. In dragonflies, the compound eyes consist of approximately 30,000 ommatidia, making them one of the species with the largest number of ommatidia among insects. These densely packed and relatively large ommatidia enable dragonflies to capture moving prey with exceptional accuracy [20,21]. Additionally, the internal structure of insect compound eyes can change in response to varying environmental conditions. When light intensity fluctuates, species such as *Ectropis grisescens* [22], *Cnaphalocrocis medinalis* [23], and *Encarsia formosa* [24], regulate the vertical movement of pigment granules by adjusting the opening and closing of the crystalline cone cells in their compound eyes. This adaptation allows them to thrive in environments differing in light intensities.

Although adult *L. xylina* exhibits typical sexual dimorphism both in phototactic and flight abilities [13], the ultrastructure of its compound eyes remains unclear. In this work, the external morphology and internal structure of the visual system of *L. xylina* were examined by scanning and transmission electron microscopy. The primary goal was to elucidate the intrinsic physical mechanisms underlying insect phototactic behavior, offering theoretical insights to aid in the development of innovative pesticide light traps for the conservation of shelter forests.

## 2. Materials and Methods

### 2.1. Insects

Pupae of the casuarina moth, *L. xylina*, were collected from Yuzhuang Village in Aodong Town (E 119°45′26″, N 25°24′39″), Pingtan County, Fuzhou City, Fujian Province, China. Individual pupae were maintained in a transparent plastic container (20 cm × 20 cm × 15 cm) under relatively constant conditions (temperature: 26 °C ± 2 °C; humidity: 70% ± 10%; photoperiod: 12L:12D). Upon eclosion, adult moths were collected and kept separately to prevent mating. The adults were provided with a 10% honey solution for one day prior to analysis. The body length and weight of each individual were recorded. A total of twelve adults were utilized in this study: six individuals (3 males and 3 females) for scanning electron microscopy (SEM) analysis, six individuals (3 males and 3 females) for transmission electron microscopy (TEM) analysis, and three two-day-old females for paraffin section preparation.

### 2.2. Light Exposure and Dark Adaptation Protocols

Adult moths were subjected to dark adaption for 2 h in a completely dark environment prior to light exposure. The light exposure was administered using a 365 nm UV lamp at an intensity of 1000 lux for 1 h from a distance of 15 cm. The exposure was conducted under controlled environmental conditions, with temperature at 26 ± 2 °C and humidity at 70 ± 10%. Ultraviolet (UV) light at 365 nm was selected for exposure experiments because previous phototaxis assays established that both male and female *L. xylina* exhibit peak attraction to this wavelength under field conditions, with males demonstrating particularly pronounced responses.

### 2.3. Scanning Electron Microscopy Compound Eye Preparation

Six adult specimens (3 males and 3 females) were selected. Heads were promptly removed and fixed overnight in 2.5% glutaraldehyde at 4 °C to ensure minimal damage during extraction. Following fixation, samples were rinsed twice with PBS for 10 min each, then subjected to secondary fixation in 1% osmium tetroxide at 4 °C for one hour. Another set of PBS rinses was performed twice for 10 min each. Prior to dehydration, the heads were carefully rinsed in phosphate-buffer saline (PBS) and briefly sonicated at low power (≤30 s) to remove surface particulates; each specimen was subsequently examined under a stereomicroscope to confirm that no surface damage occurred. Samples were dehydrated through a graded ethanol series (30%, 50%, 70%, 80%, 90%, 95%, and absolute ethanol), with each concentration applied twice for 15 min. Following absolute ethanol dehydration, specimens were transferred to a 1:1 mixture of acetone and isoamylacetate for 10 min as a transition fluid. Specimens were then dried using a critical point dryer (Autosamdri-815A, Tousimis Research Corporation, Rockville, MD, USA) and securely mounted with conductive adhesive. The samples were then coated with a thin layer of gold via sputter coating under vacuum conditions (10 kV, 220 s). Finally, prepared samples were carefully examined by SEM (Hitachi SU8020, Hitachi High-Technologies, Tokyo, Japan).

### 2.4. Transmission Electron Microscopy Compound Eye Preparation

Three adult specimens of each sex were selected and immobilized by freezing. Heads were immediately removed to expose the compound eyes. The fixation and dehydration procedures followed those established for SEM (Section 2.2), with the following modifications: specimens underwent three 15 min PBS rinses (rather than two 10 min rinses), osmium tetroxide postfixation was extended to 2 h (rather than 1 h), and ethanol dehydration was conducted in a single 15 min step per concentration (rather than two steps). Following dehydration, the samples were infiltrated overnight with acetone-resin mixtures (3:1 for 2 h, 1:1 for 3 h, and 1:3 for 3 h), embedded in molds, and polymerized using a temperature gradient (35 °C for 5 h, 60 °C for 5 h, and 80 °C for 5 h). Ultrathin sections (70–90 nm) were cut using a LEICA EM UC7 (Leica Microsystems, Vienna, Austria) ultramicrotome, stained with uranyl acetate for 15 min and lead citrate for 5 min, and air-dried. Sections were examined using a tungsten filament TEM (JEM-1200EX, JEOL Ltd., Tokyo, Japan).

### 2.5. Histological Preparation

Heads of two-day-old female *L. xylina* adults were collected and sonicated in phosphate-buffered saline (PBS) to remove surface contaminants. Specimens were fixed in Bouin’s fixative at 25 °C for 24 h. Following fixation, tissues were dehydrated through an ascending ethanol series (75%, 85%, 90%, 95%, and absolute ethanol), with 60 min immersions at each concentration. Clearing was performed sequentially in ethanol-xylene (5–10 min) followed by xylene I (5–10 min) and xylene II (5–10 min). Tissues were then infiltrated with paraffin at 65 °C through three successive one-hour incubations. Embedding was performed using a JB-P5 embedding system (JB-P5, Wuhan Junjie Electronics Co., Ltd., Wuhan, China), and blocks were rapidly cooled on a −20 °C cold plate (JB-L5). After paraffin solidification, tissue blocks were trimmed. Serial sections (4 μm) were cut using an RM2016 rotary microtome (RM2016, Leica Microsystems, Shanghai, China), floated on 40 °C distilled water (KD-P), and mounted onto glass slides. Histological slides were incubated at 60 °C (GFL-230 incubator, Gesellschaft für Labortechnik mbH, Burgwedel, Germany) to ensure section adhesion and complete desiccation. Sections underwent clearing in xylene I and II (10 min each), followed by rehydration through a graded ethanol series (absolute ethanol, 95%, 90%, 85%, and 75%; 2–3 min per concentration). Subsequently, sections were stained with hematoxylin for 5–8 min. Slides were then rinsed in running tap water for 10 min, differentiated in 1% hydrochloric acid ethanol for 30 s, blued in Scott’s tap water substitute for 3 min, and counterstained with eosin for 1–2 min. Following staining, sections were dehydrated through an ascending ethanol series (75%, 85%, 90%, 95%, and absolute ethanol; 2–3 min per step), cleared in xylene I and II (5 min each), and permanently mounted with neutral balsam. Sections were dewaxed in xylene, rehydrated through a descending ethanol series, and stained with hematoxylin and eosin (H&E). Following final dehydration and clearing, slides were permanently mounted with neutral balsam. Slices were observed using a panoramic scanner (FLS-PRO4, Thermo Fisher Scientific, Waltham, MA, USA).

### 2.6. Data Analysis

High-resolution images were processed using Adobe Photoshop 2020. The projected compound eye area and perimeter were quantified from frontal-view SEM images using two-dimensional projections. Individual compound eye outlines were manually delineated in ImageJ 1.54f utilizing the freehand selection tool. Subsequent to calibration with SEM scale bars, the software computed the corresponding projected area and perimeter values. The number and area of ommatidia were also quantified using ImageJ. Histological measurement data of compound eyes between male and female adults were statistically evaluated using independent sample *t*-tests with SPSS 16.0 software (significance levels set at *p* < 0.05 and *p* < 0.01). Panoramic observation and annotation of paraffin sections were performed using SlideViewer software 2.6.0.

## 3. Results

### 3.1. External Morphology of Compound Eyes in L. xylina

**Figure 1:**
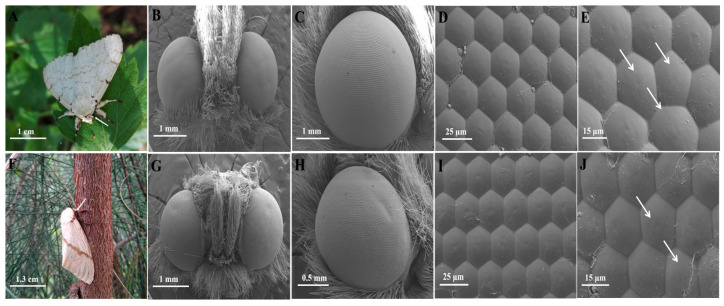
The external morphology of the compound eyes of male and female L. *xylina*. (**A**) Male casuarina moth (arrow indicating the compound eyes). (**B**) Ventral view of the male compound eyes. (**C**) Lateral view of the male compound eyes. (**D**) Hexagonal ommatidia of the male compound eyes. (**E**) Irregular ommatidia (arrows indicating) of the male compound eyes. (**F**) Female casuarina moth (arrow indicating the compound eyes). (**G**) Frontal view of the female compound eyes. (**H**) Lateral view of the female compound eyes. (**I**) Hexagonal ommatidia of the female compound eyes. (**J**) Irregular ommatidia (arrows indicating) of the female compound eyes.

Adult *L. xylina* exhibit sexual dimorphism in body size, with females being significantly larger (body length: 2.59 ± 0.23 cm; body weight: 0.69 ± 0.25 g) than males (body length: 2.02 ± 0.25 cm; body weight: 0.19 ± 0.05 g). In both sexes, the compound eyes are located laterally on the head and exhibit smooth surfaces and a parallel-symmetrical arrangement (Figure 1).

Male compound eyes had an average long axis of 1.86 ± 0.06 mm and a short axis of 1.60 ± 0.02 mm (Figure 1B,C), whereas female compound eyes had an average long axis of 1.68 ± 0.03 mm and a short axis of 1.47 ± 0.05 mm (Figure 1G,H). The calculated axis ratios were 1.16 in males and 1.14 in females, and, in gross morphology both sexes presented an oval contour, together indicating that the overall eye shapes of males and females were very similar. In both sexes, the ommatidia exhibited a tightly packed and regular hexagonal arrangement, with occasional pentagonal or slightly irregular facets observed (Figure 1E,J).

Compared to females, male adults possessed significantly larger compound eye area, perimeter, ommatidia number, facet area, and facet perimeter (*p* < 0.05). However, there is no significant difference in ommatidia spacing between males and females (*p* > 0.05). The average compound eye area of the male (2.67 ± 0.09 mm^2^) is 44.32% larger than that of the female (1.85 ± 0.02 mm^2^), and the compound eye circumference of the male (6.54 ± 0.12 mm) is 35.12% longer than that of the female (4.84 ± 0.05 mm). The male has approximately 10% more ommatidia (7752.83 ± 119.87) than the female has(7062.17 ± 42.33). The ommatidia area (facet area) of the male (562.79 ± 9.66 μm^2^) is extremely significantly larger than that of the female (356.4 ± 64.63 μm^2^). The ommatidia circumference of the male (91.83 ± 1.29 μm) is 23.07% longer compared to that of the female (74.62 ± 0.94 μm) (Table 1).

### 3.2. Internal Structure of the Compound Eyes in L. xylina

**Figure 2:**
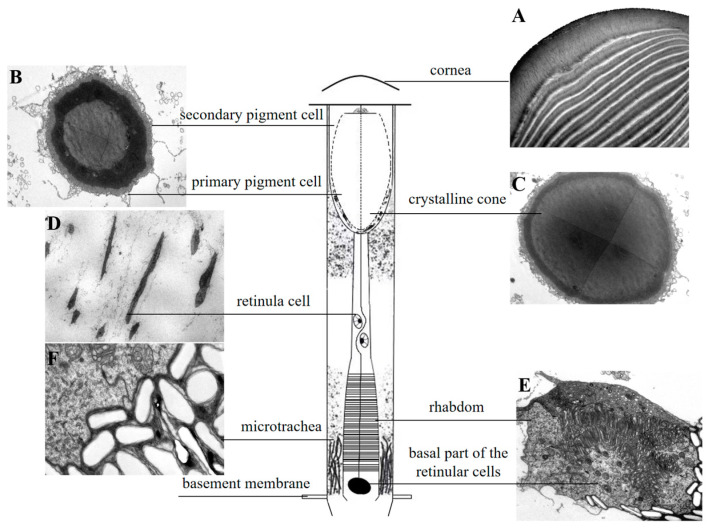
Schematic diagram of the longitudinal section of the ommatidium in *L. xylina.* (**A**) Longitudinal section of the cornea. (**B**) Cross-section showing the crystalline cone surrounded by primary and secondary pigment cells. (**C**) Cross-section of the crystalline cone. (**D**) Longitudinal section of a retinula cell. (**E**) Cross-section of the rhabdom (with an additional basal membrane cell compared to retinula cells). (**F**) Cross-section of the micro-trachea.

The compound eye of *L. xylina*, features a distinct “clear zone”, and is of the superposition type, showing no sexual structural differences. The eye structure, from the outermost to the innermost layer, comprises the cornea, crystalline cone, primary and secondary pigment cells, clear zone, rhabdom, and basal layer, as illustrated in the diagram (Figure 2).

**Figure 3:**
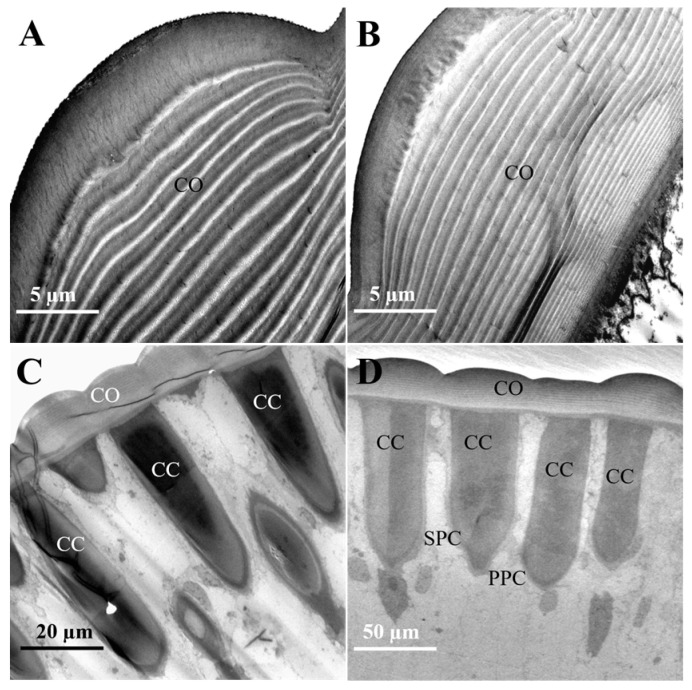
The cornea and crystalline cone in the distal ommatidial region of female and male *L. xylina*. (**A**) Longitudinal section of the cornea in the female. (**B**) Longitudinal section of the cornea in the male. (**C**) Longitudinal section of the crystalline cone in the female. (**D**) Longitudinal section of the crystalline cone in the male. CO: cornea; CC: crystalline cone; PPC: primary pigment cell; SPC: secondary pigment cell.

The cornea, the outermost layer of the ommatidium (Figure 3A,B), is significantly thicker in females (16.3 ± 0.41 μm) compared to in males (13.43 ± 0.53 μm). Corneal width shows no significant difference between sexes (females: 19.92 ± 1.77 μm; males: 19.86 ± 1.63 μm). Within the cornea, the outer layers are arranged more loosely, whereas the inner layers are more densely packed towards the center. The cornea of male moths consists of approximately 25 ± 1.15 layers, and that of female moths has about 28 ± 0.58 layers. There is no significant difference in layer count between the sexes (Figure 3A,B and Table 2).

Situated beneath the cornea, the crystalline cone functions with the cornea to form the optical system, focusing light onto the photoreceptor cells. In females, the crystalline cones measured 57.99 ± 1.29 μm in length and 13.66 ± 0.70 μm in width (Figure 3C). In some sections, droplet-like profiles appeared separated from the cornea. The observed separation may result from sectioning artifacts and was excluded from measurements. In contrast, male adults possess cylindrical crystalline cones that attach to the cornea in longitudinal sections, measuring 61.48 ± 1.19 μm in length and 12.33 ± 1.44 μm in width (Figure 3D).

The inter-ommatidial angle is 3.49 ± 0.27° for males and 3.62 ± 0.25° for females; there is no significant differences in the angle between the sexes (Table 2).

**Figure 4:**
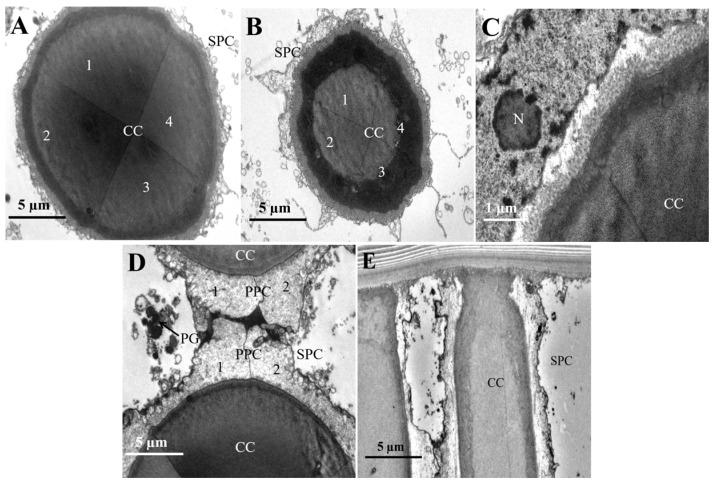
The micro-graph of the crystalline cone in the compound eyes of female *L. xylina*. (**A**) Cross-section of the crystalline cone. (**B**) Cross-section of the crystalline cone. (**C**) Edge of the crystalline cone. (**D**) Tip of the crystalline cone. (**E**) A pair of primary pigment cells surrounds the crystalline cone. CC: crystalline cone; PPC: primary pigment cells; SPC: secondary pigment cell; PG: pigment granule. N: nucleus.

The crystalline cone exhibits a circular cross-section and is composed of four crystalline cone cells and their secretions, classifying it as eucone (Figure 4A,B). A pair of primary pigment cells surround the crystalline cone, while secondary pigment cells and pigment granules are located at both the periphery and the tip (Figure 4D,E).

**Figure 5:**
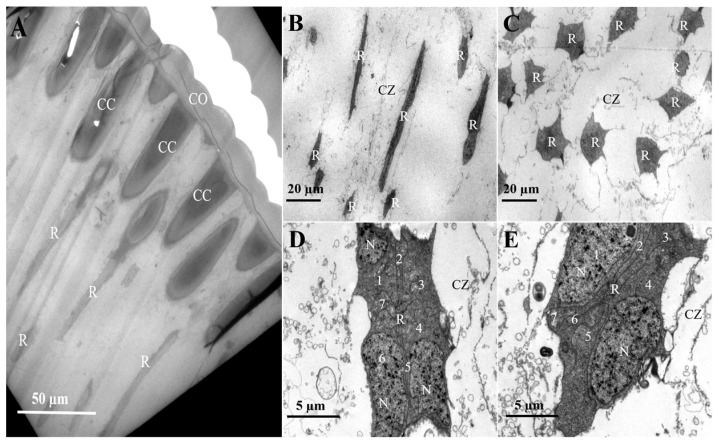
Retinula cell arrangement in the compound eyes of female *L. xylina*. (**A**) Longitudinal section of the compound eye (**B**) Longitudinal section of the retinula cell. (**C**) Cross-section of the retinula cell. (**D**) Distal end of the retinula cell. (**E**) Proximal end of the retinula cell. CO: cornea; CC: crystalline cone; R: retinula cell; CZ: clear zone; N: nucleus.

Positioned subjacent to the crystalline cone, the clear zone contains retinula cells with distinct planar morphologies. Retinula cells exhibit linear alignment along the ommatidia axis (Figure 5B) and adopt a concentric radial arrangement around a central axis (Figure 5C). They are grouped into sets of seven, and no specialized micro-villi were observed on their inner surfaces, indicating they do not form rhabdoms at this part (Figure 5D). Proximally, in the region of the retinal cell nuclei, the space between adjacent retinal cell bundles narrows and arranges radially. At this level, however, the seven retinula cells have not yet formed a rhabdom (Figure 5E).

**Figure 6:**
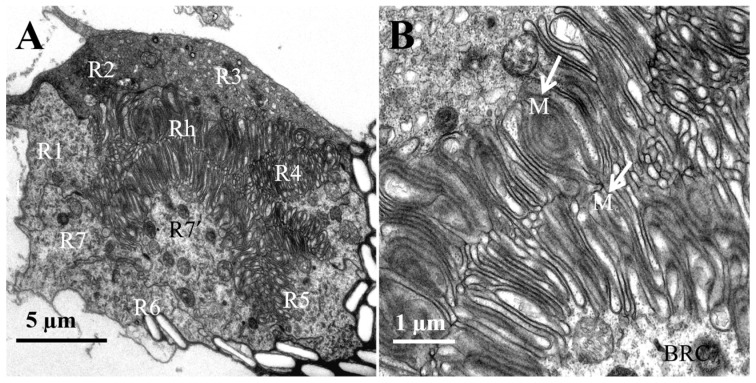
TEM micro-graph of the region of rhabdoms in the female *L. xylina*. (**A**) Cross-section of the rhabdom. (**B**) Micro-villi (indicated by arrows). Rh: rhabdom; BRC: basal part of the retinular cells; M: micro-villi.

The basal part of the retinula cells is present at the base of the ommatidium, centrally surrounded by the other seven retinula cells, forming a closed rhabdom (Figure 6A). The inner membrane of the basal cell is specialized into numerous micro-villi that aggregate to form the rhabdom (Figure 6B).

**Figure 7:**
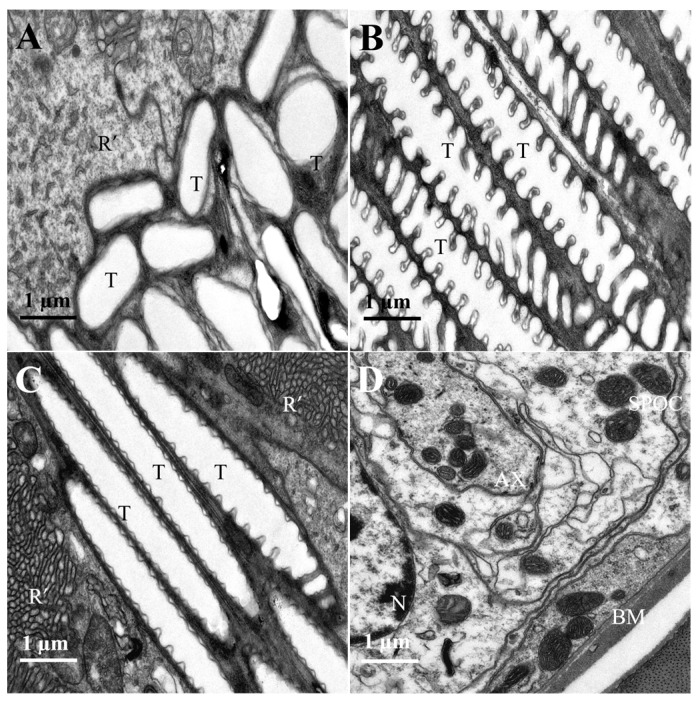
Proximal region of the ommatidial retina of female *L. xylina*. (**A**) Longitudinal section of the micro-trachea (distal end). (**B**) Longitudinal section of the micro-trachea (proximal end). (**C**) Longitudinal section of the micro-trachea (proximal end). (**D**) Cross-section of the basal membrane. R’: rhabdomere; T: micro-trachea; N: nucleus; AX: axon; BM: basal membrane; SPOC: subretinular pigment cell.

Numerous micro-tracheae are present below the rhabdoms at the base of the compound eye, exhibiting a ridge-like structure. Their morphology varies spatially, appearing irregularly elliptical at the distal end (Figure 7A) and elongated and rugged at the proximal end (Figure 7B,C). The structure at the base of the ommatidium, known as the basal membrane (Figure 7D), acts as the supporting framework for the compound eye. It separates the photoreceptive layer from the neural layer, ensuring the stability and overall shape of the eye.

**Figure 8:**
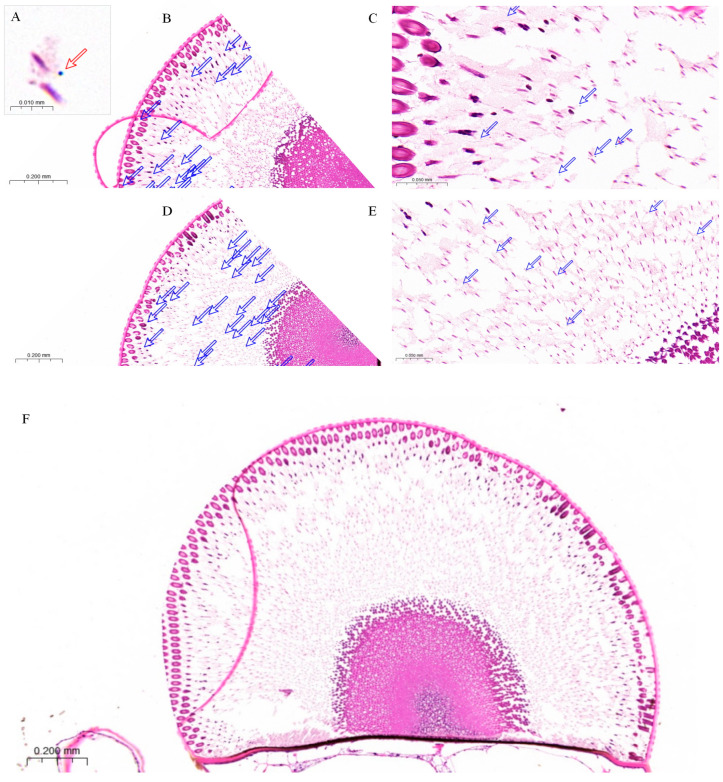
Paraffin section of the compound eye of female *L. xylina*. (**A**) Pigment granule (indicated by red arrow). (**B**) Distribution of pigment granules (pink dots and indicated by arrows) in the compound eye after dark adaptation. (**C**) Pigment granules localized around and beneath the crystalline cones under dark conditions. (**D**) Distribution of pigment granules in the compound eye under 365 nm light exposure. (**E**) Pigment granules under light exposure, gradually migrating toward the rhabdoms. (**F**) Paraffin section of the compound eye of the female moth after exposure to 365 nm ultraviolet light.

The spatial distribution of pigment granules within the compound eyes of two-day-old female *L. xylina* exhibited pronounced redistribution under varying illumination regimes. Following dark adaptation, pigment granules were predominantly localized peripheral to the crystalline cones (Figure 8B,C). Conversely, after 60 min of exposure to 365 nm light, the granules progressively migrated towards the rhabdom region (Figure 8D–F).

## 4. Discussion

The sexual differences in the compound eye structure of *L. xylina* are likely related to the distinct visual needs of males and females. Males, which exhibit active flight in search of mates, possess larger compound eye circumferences (6.54 ± 0.12 mm) and a greater number of ommatidia (7752.83 ± 119.87) compared to females. This adaptation enhances their ability to capture light, likely facilitating mate-searching behavior in low-light environments. In contrast, females are largely stationary, waiting for males, and thus have lower visual demands, leading to a smaller compound eye (4.84 ± 0.05 mm) and fewer ommatidia (7062.17 ± 42.33) [25]. This is consistent with previous research on compound eye structures in other Lepidoptera species, including *Diaphania glauculalis* [26], *Eutectona machaeralis* [27], *Orgyia antiqua* [28], *Ectropis grisescens* [22], *Operophtera brumata* [29], and *Athetis lepigone* [30].

The inter-ommatidial angle of *L. xylina* (average 3.54°) was relatively large compared to diurnal insects like Odonata (0.24–1.2°), which are recognized for possessing the highest visual acuity among insects. The inter-ommatidial angle of insects directly governs the spatial resolution of their compound eyes and is intricately linked to both the extent of visual field overlap and the breadth of the visual field [31,32,33]. The absence of significant sexual dimorphism in the inter-ommatidial angle suggests that males achieve a wider field of view and enhanced light sensitivity under low-light conditions through an increase in ommatidial area and count, rather than via improved resolution. Therefore, the visual advantage of male *L. xylina* appears more related to their ability to adapt to low-light conditions rather than to high resolution.

Our findings indicate that the compound eyes of *L. xylina* exhibit a parallel symmetrical arrangement with smooth corneal surfaces. Both eyes are similar in morphology and size, confirming their symmetry. This finding is consistent with previous research on other nocturnal insects, including the *Grapholita molesta* [34] and the *Ostrinia furnacalis* [35]. The symmetrical structure of the compound eyes minimizes potential structural differences arising from asymmetric eye development, thereby ensuring balanced light exposure, reducing visual blind spots, and enhancing the overall function of the visual system. As a typical nocturnal insect, *L. xylina* lives in low-light environments and requires a wide field of view and high light sensitivity. These adaptations help maintain balanced visual input, reducing the brain’s load for image correction and integration and thus improving both response speed and energy efficiency.

The arrangement of ommatidia in insect compound eyes plays a vital role in shaping their ecological niche. According to Friedrich et al. [36], in *L. xylina*, each ommatidium comprises seven distal retinula cells and one basal retinula cell, exhibiting a “7 + 1” configuration, which is also found in species such as the *Grapholita molesta* [34] and the *Acleris fimbriana* [37]. In contrast, species in the *Childrena zenobia* [38], *Neptis beroe* [38], *Cephonodes hylas* [39], and *Cechenena lineosa* [39] follow an “8 + 1” arrangement, while the *Eutectona machaeralis* [27] and the *Ostrinia furnacalis* [35] display an “11 + 1” pattern, and the *Ectropis grisescens* [22] has a “14 + 1” arrangement. Studies have shown that the arrangement of retinula cells is species-specific, as evidenced by the distinct configurations observed in various insect species. Furthermore, the number and arrangement of retinula cells in insect ommatidia varies significantly across species (Table 3)..

The variation in retinula cells highlights the diversity in phenotypic plasticity across species adapted to different environments. The internal structure of the compound eye plays a crucial role in determining insect behavior [33]. Insects generally exhibit two distinct types of compound eyes: apposition eyes and superposition eyes. Most diurnal insects, such as bees and wasps within Hymenoptera, possess apposition compound eyes adapted for high spatial resolution under bright illumination, while numerous nocturnal insects—including moths and certain coleopterans—exhibit refracting superposition eyes. In superposition eyes, light from multiple ommatidia converges optically through a “clear zone” constituted by the cytoplasmic transparency of secondary pigment cells. This mechanism enhances photosensitivity at the cost of reduced spatial resolution, thereby facilitating visual function in dimly illuminated or open habitats [26,28,40]. Notably, nocturnal Hymenoptera do not employ superposition optics; even night-flying bees retain apposition eyes and compensate for low-light conditions through enlarged facets or neural summation rather than optical superposition [41]. Coleoptera exhibits remarkable diversity in ocular morphology: nocturnal species possess superposition eyes analogous to those of moths, whereas particularly burrowing or ground-dwelling species feature apposition eyes with unusually thickened corneal lenses that afford protection against mechanical damage and intense illumination [15]. Hemiptera likewise spans a spectrum of visual ecologies: fast-moving predatory bugs typically have relatively large, acute compound eyes for prey tracking, whereas sedentary sap-feeders (e.g., scale insects or aphids) rely less on vision and may exhibit fewer ommatidia or even vestigial eyes [42,43]. Correspondingly, in Lepidoptera insects, moths and butterflies have completely different living habits and hunting strategies [36], thus evolving compound eye structures with different functions. Butterflies typically have nine photoreceptors [44,45,46], while those in the moth family only have eight photoreceptors, as demonstrated by *L. xylina* in this study and *Orgyia antiqua* [28].Together, these cross-order comparisons underscore both the conserved function of the compound eye and its evolutionary flexibility: similar environmental pressures (e.g., low-light nocturnal conditions or visually guided predation) have repeatedly produced functionally analogous adaptations in different lineages, while distinct life histories have driven unique modifications in eye structure and size.

In apposition eyes, the ommatidia are aligned in parallel rows, thereby offering enhanced resolution to the insect’s visual field. This arrangement allows for more precise localization and recognition of targets. Insects like *Terrobittacus implicatus* [47] and *Meimuna mongolica* [48] are typical examples of those with apposition eyes. This study revealed a distinct, clear zone between the crystalline cone and rhabdom within the compound eyes of *L. xylina*, suggesting their possession of superposition eyes.

In the compound eyes of adult *L. xylina*, seven retinula cells extend through the clear zone towards the crystalline cone; however, cells lack micro-villi in this region and therefore do not form rhabdoms at this level, apart from the basal cell. This observation aligns with the compound eye structure of the *Orgyia antiqua* [28] in the subfamily Lymantriinae. At the base of the compound eye, clustered micro-tracheae form a reflective tracheal layer. The layer likely helps to adjust reflected light and prevents light scattering or crosstalk between adjacent rhabdoms. As a result, it not only enhances the light sensitivity of the compound eyes but also contributes to efficient light detection and improved signal clarity in adult *L. xylina*.

In many nocturnal insects, pigment granules within the clear zone of the retina play a crucial role in absorbing and filtering light [49,50]. These granules typically shift in response to variations in light intensity and direction, controlling the amount of light entering the ommatidium. Under intense light conditions, the granules may accumulate in the transparent regions of the cells, thereby reducing light transmission and protecting the retina from potential damage [24,51].

Paraffin sections of compound eyes of *L. xylina* revealed that, under dark-adapted conditions, pigment granules were primarily localized adjacent to the crystalline cones. This distribution presumably expands the photosensitive region, thereby augmenting the moth’s sensitivity to low-intensity light stimuli. Conversely, exposure to intense 365 nm ultralight elicited the inward migration of the pigment granules towards the rhabdoms, forming an effective light-shielding structure. This configuration minimizes photic interference with photoreceptor cells, confers protection against light-induced cellular damage, and facilitates visual adaptation under high-intensity illumination. These findings align with established light and dark adaptation mechanisms in Lepidopteran insects [34,52], indicating that *L. xylina* achieves rapid functional adjustment to variable light environments through pigment granule migration.

In this study, the eye morphology of *L. xylina* was examined using scanning and transmission electron microscopy (SEM/TEM) and histological sectioning, which provide high-resolution details of surface and cellular structures. As a complementary approach, micro-computed tomography (micro-CT), including advanced synchrotron-based forms, allows non-destructive three-dimensional visualization of compound eye architecture and avoids sectioning artifacts [41,53]. This technique has been successfully applied to reveal the spatial organization of ommatidia, lenses, and optic nerves even in very small insects [54], although its resolution remains lower than that of electron microscopy and thus cannot resolve ultrastructural details such as photoreceptor micro-villi [55]. By contrast, SEM/TEM and histology excel at resolving fine features but are labor-intensive and restricted to two-dimensional slices. In practice, these approaches are complementary: micro-CT enables holistic 3D reconstructions and morphological quantification [56], whereas electron microscopy remains indispensable for ultrastructural confirmation. Together, they provide a comprehensive framework for understanding insect eye morphology.

## 5. Conclusions

In conclusion, this study demonstrates that the compound eyes of *L. xylina* are characteristic superposition eyes, exhibiting marked sexual dimorphism in their external morphology. Male individuals possess significantly larger compound eyes, likely linked to a heightened need for visual sensitivity in mate-searching. Although the ultrastructural composition of the compound eyes is largely similar between males and females, distinct morphological differences remain, particularly in corneal thickness and crystalline cone length. This sexual dimorphism in visual organs is likely associated with the distinct ecological adaptation strategies of *L. xylina*. Furthermore, paraffin sectioning demonstrated the regional heterogeneity and dynamic redistribution of pigment granules, indicating that the compound eyes exhibit photoadaptive properties through visual modulation mechanisms. Our findings highlight the pivotal role of sensory morphology in ecological adaptation, providing a reference for optimizing sex-specific international quarantine surveillance and offering a morphological basis for the development of light-trapping techniques in nocturnal insects.

## Figures and Tables

**Table 1:** Comparison of external morphological properties of the compound eyes between male and female *L. xylina*.

Parameter	Gender		*p*-Value
	Female	Male	
Compound eye area (mm^2^)	1.85 ± 0.02	2.67 ± 0.09	<0.001
Compound eye perimeter (mm)	4.84 ± 0.05	6.54 ± 0.12	<0.001
Facet number per eye	7062.17 ± 42.33	7752.83 ± 119.87	0.014
Facet area (μm^2^)	356.4 ± 64.63	562.79 ± 9.66	0.002
Facet perimeter (μm)	74.62 ± 0.94	91.83 ± 1.29	<0.001
Facet spacing (μm)	0.45 ± 0.06	0.57 ± 0.03	0.124

Data are presented as mean ± standard error. A significance level of (*p* < 0.05) was considered significant, and (*p* < 0.01) was considered highly significant.

**Table 2:** Comparison of the internal structure of the compound eyes between male and female adult *L. xylina*.

Parameter	Gender		*p*-Value
	Female	Male	
Crystalline cone width (μm)	13.66 ± 0.70	12.33 ± 1.44	0.610
Crystalline cone length (μm)	57.99 ± 1.29	61.48 ± 1.19	0.026
Corneal thickness (μm)	16.3 ± 0.41	13.43 ± 0.53	0.002
Corneal width (μm)	19.92 ± 1.77	19.86 ± 1.63	0.981
Interommatidial angle (°)	3.62 ± 0.25	3.49 ± 0.27	0.725
Number of Corneal Layers	28.00 ± 0.58	25.00 ± 1.15	0.079

Data are presented as mean ± standard error. A significance level of (*p* < 0.05) was considered significant, and (*p* < 0.01) was considered highly significant.

**Table 3:** Number of retinula cells in the compound eyes of insects from different families.

Family	Insect	Distal Retinula Cells	Basal Retinula Cells	References
Geometridae	*Ectropis grisescens*	14	1	[22]
	*Operophtera brumata*	14	1	[29]
Pyralidae	*Eutectona machaeralis*	11	1	[27]
	*Ostrinia furnacalis*	11	1	[35]
Nymphalidae	*Childrena zenobia*	8	1	[38]
	*Neptis beroe*	8	1	[38]
Sphingidae	*Cephonodes hylas*	8	1	[39]
	*Cechenena lineosa*	8	1	[39]
Tortricidae	*Grapholita molesta*	7	1	[34]
	*Acleris fimbriana*	7	1	[37]

## Data Availability

The original contributions presented in this study are included in the article. Further inquiries can be directed to the corresponding authors..
